# Spatial metabolomics in head and neck tumors: a review

**DOI:** 10.3389/fonc.2023.1213273

**Published:** 2023-07-13

**Authors:** Ye Zheng, Chen Lin, Yidian Chu, Shanshan Gu, Hongxia Deng, Zhisen Shen

**Affiliations:** ^1^ Health Science Center, Ningbo University, Ningbo, China; ^2^ The Affiliated Lihuili Hospital, Ningbo University, Ningbo, China

**Keywords:** Spatial metabolomics, head and neck tumors, tumor metabolism, metabolic reprogramming, mass spectrometry imaging

## Abstract

The joint analysis of single-cell transcriptomics, proteomics, lipidomics, metabolomics and spatial metabolomics is continually transforming our understanding of the mechanisms of metabolic reprogramming in tumor cells. Since head and neck tumor is the sixth most common tumor in the world, the study of the metabolic mechanism of its occurrence, development and prognosis is still undeveloped. In the past decade, this field has witnessed tremendous technological revolutions and considerable development that enables major breakthroughs to be made in the study of human tumor metabolism. In this review, a comprehensive comparison of traditional metabolomics and spatial metabolomics has been concluded, and the recent progress and challenges of the application of spatial metabolomics combined multi-omics in the research of metabolic reprogramming in tumors are reviewed. Furthermore, we also highlight the advances of spatial metabolomics in the study of metabolic mechanisms of head and neck tumors, and provide an outlook of its application prospects.

## 1 Introduction

Spatial metabolomics (1) is a novel molecular imaging technology based on mass spectrometry imaging (MSI) (2) technology. The metabolite on the tissue sections can be collected and detected point by point through it. It can obtain information on the content and spatial distribution of numerous molecules such as endogenous metabolites and exogenous drugs directly from biological tissues, thus achieving high spatial resolution and accurate positioning of the metabolite distribution in the tissue. Consequently, spatial metabolomics is essential to elucidate the mechanism of the synthesis, accumulation, and regulation of metabolites.

Tumors are an increasingly serious public health problem worldwide (3), and statistics have shown that one in six women and one in five men in the world develop tumors throughout their lifetime. Despite increasingly advanced methods of early diagnosis and corresponding treatments resulting in improved patient survival time, the mortality rate of tumor patients remains high.

In the past few decades, scientists have made great progress in studying the occurrence and development of tumors and the mechanism of tumor metabolism (4). The occurrence and development of tumors require tumor cell metabolic reprogramming (5). By autonomously changing the flux of various metabolic pathways, tumor cells can meet the raising bioenergy and biosynthesis needs of tumor tissue, and also alleviate and adapt to the oxidative stress required for tumor cell proliferation and survival. A growing body of evidence supports that tumor metabolic reprogramming not only plays a crucial role in maintaining the cell signaling pathways for tumor occurrence and survival but also has broader significance in regulating anti-tumor immune responses by releasing metabolites to affect the expression of immune molecules such as prostaglandin (PG) (6).And the metabolic heterogeneity of tumors (7) is an important part of tumor heterogeneity, which specifically refers to the significant differences in the metabolic characteristics of different tumors or within the same tumor tissue. Therefore, due to the diversity, complexity, and heterogeneity of tumor tissues, we must analyze several different tissue samples and metabolites to explore the underlying mechanisms of the occurrence, development, proliferation, and metastasis of tumors, which has become a major obstacle limiting research on tumor metabolic mechanisms (8). Benefiting from improvements in the imaging resolution and detection sensitivity of the mass spectrometer, spatial metabolomics can accurately measure the type, content, and spatial distribution of metabolites in human tissues. This helps in overcoming a longstanding resolution obstacle, expanding the dimensions of metabolome information, and greatly improving the metabolite information depth of tissue samples.

In this review, we mainly summarize the development from traditional metabolomics to spatial metabolomics combined with multi-omics analysis, demonstrate the comparison of the applications of different platforms of spatial metabolomics, and discuss the data-mining challenges that spatial metabolomics analysis is facing nowadays. In addition, we orderly review the novel applications of spatial metabolism combined with multi-omics analysis in the study of tumor metabolism mechanism, and also focus on its latest research in head and neck tumors. Then we end with the prospects for its application in the diagnosis and treatment of head and neck tumor metabolism.

## 2 Traditional metabolomics and spatial metabolomics

**Figure 1:**
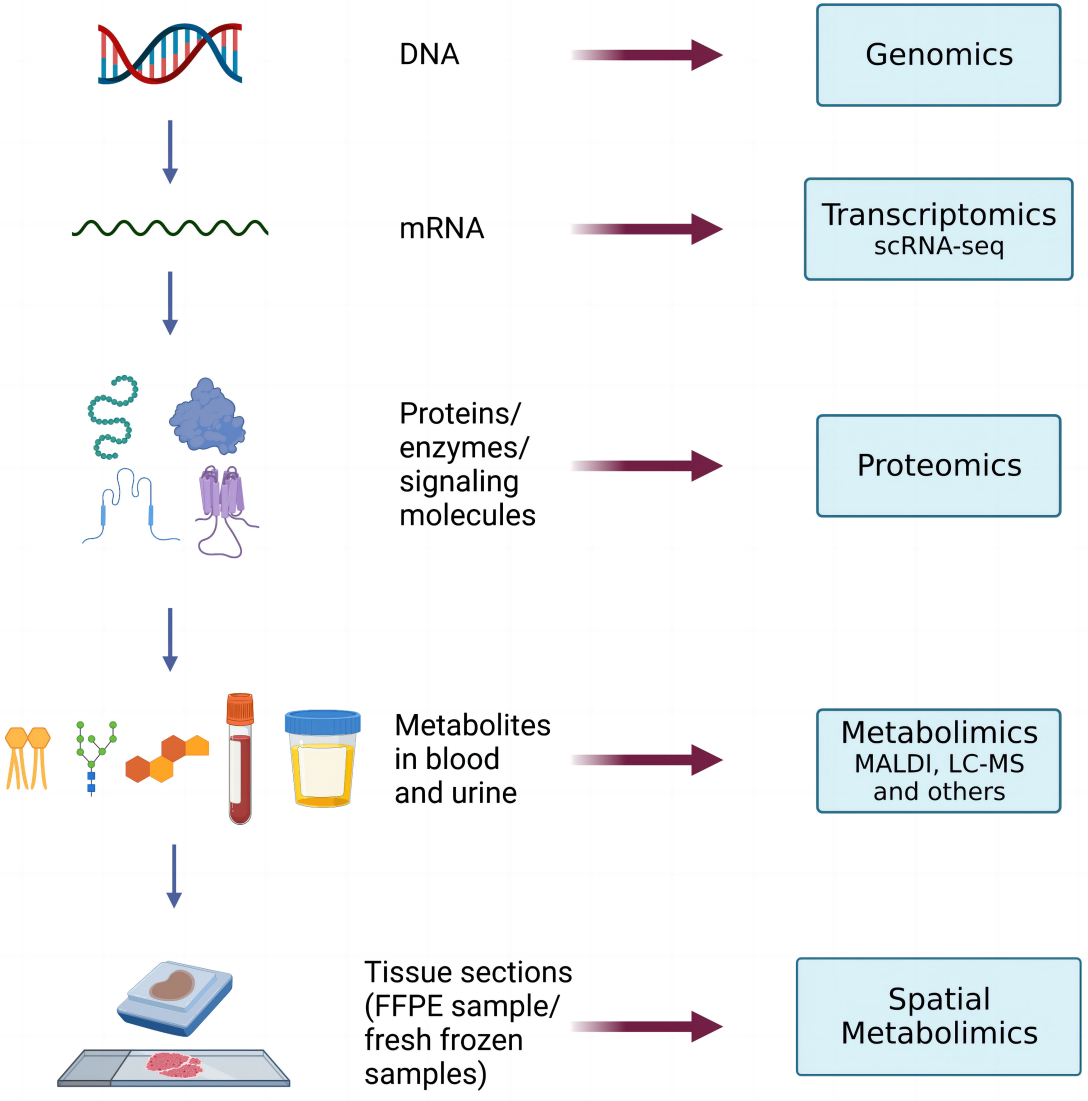
Manufacturing flow for the final spatial metabolic images from the tumor samples.

Spatial metabolomics integrates MSI and metabolomics (9) technology. By using a mass spectrometry imager, metabolites on tissue sections can be collected and detected point by point. We can then accurately identify and locate the differential distribution of various metabolites among tissues and conduct in-depth metabolomic analyses on the target micro-area tissues. Finally, we can restore the detected metabolites at each point to a two-dimensional level so that the qualitative, quantitative, and localized information of small molecule metabolites can be obtained at once. (**Figure 1**)

MSI is a new imaging method that directly scans biological samples through mass spectrometry technology. It possesses the advantages of no labeling, multipoint detection, and high sensitivity, and it can simultaneously analyze the spatial distribution characteristics of hundreds of molecules on the same tissue section. With the continuous development of modern science and technology, and meeting deeper scientific research needs, various MSI types have been developed, including matrix-assisted laser/desorption ionization mass spectrometry imaging technology (MALDI-MSI) (10), desorption electrospray ionization mass spectrometry imaging (DESI-MSI) (11), 3D imaging — secondary ion mass spectrometry (SIMS) (12) and so on. Among them, airflow-assisted desorption and electrospray ionization mass spectrometry imaging (AFADESI-MSI) (13) has recently acquired considerable popularity and extensive applications (14, 15). This is an environmental molecular imaging technique with the characteristics of wide coverage, high sensitivity, wide dynamic range, high specificity, and high heterogeneity (16), which can map out a variety of functional metabolites located in different metabolic pathways.

The different platforms of MSI and their application conditions are summarized in the following table (**Table 1**). For example, MALDI-MSI possesses the advantage of high spatial resolution (17), while DESI-MSI possesses the peculiarity of high throughput and high efficiency (18). And SIMS may cause damage to the surface of the sample by the ion beam (19), but it has the characteristic of subcellular imaging (20), high spatial resolution and high efficiency.

**Table 1:** Comparison of different platforms of MSI used for spatial metabolomics.

	Spatial resolution (um)	Whether substrate is needed?	Applicable sample	Limitations	Advantages
**MALDI-MSI**	5-200	Substrate assistance is needed to help ionize the material.	Biological macromolecules (proteins, peptides and lipids)	The existence of matrix effects; The operating condition of high vacuum; Not suitable for large tissue samples	High spatial resolution
**DESI-MSI**	100-500	No.	Small molecules (metabolites and lipids)	Low spatial resolution; Low sensitivity; Not suitable for large tissue samples	Simple preparation procedure of sample; The operating condition of atmospheric pressure; High throughput; High efficiency
**AFADESI-MSI**	40-100	No. Small molecular material present on the surface of the sample can be extracted by organic reagents.	Small molecules (lipids and small molecules below 500Da)	Not suitable for large molecules such as proteins and peptides	The operating condition of atmospheric pressure; Wide range in slice size
**SIMS**	0.1-0.5	No.	Wide range	The ion beam may cause the fragmentation and the damage of the surface of the sample.	Subcellular imaging; High spatial resolution; High efficiency

The metabolome is a collection of small molecular chemical entities which can involve in metabolism and maintaining the normal growth and development of organisms (21), and the purpose of its research is to find and identify biomarkers in the diagnosis and prediction of disease. The human metabolome is affected by changes in the proteome and genome and can be interpreted as the most downstream end-product of the cellular phenotype. The metabolome affects cell physiology through regulation at the genomic, transcriptomic, and proteomic levels, among others. Metabolomics is the science that studies changes in the metabolome of biological systems (cells, tissues, or organisms) when stimulated or disturbed. Metabolomics uses advanced analytical chemical techniques to enable the high-throughput characterization of metabolites in cells, organs, tissues, or biological fluids (22, 23). The value of metabolomics has been redefined from simple biomarker identification tools as techniques for discovering driving factors of biological process activity. The development of omics techniques has better improved our understanding of the normal physiology and the pathophysiology of many diseases. It is believed that metabolomics not only reflects altered genetic and proteomic function, but also carries more information about the cellular phenotype.

Metabolomics is a powerful analytical tool for studying metabolite spectrum, metabolic changes, metabolic pathways, and the discovery of biomarkers, so the ultimate goal of studying metabolomics is to make a qualitative and quantitative analysis of all metabolites in a biological system as much as possible. Traditional metabolomics mainly relies on these three technical platforms: nuclear magnetic resonance (NMR) (24), Gas Chromatography-Mass Spectrometry (GC-MS) (25), and Liquid Chromatograph-Mass Spectrometer (LC-MS) (26, 27). The three platforms each have their advantages and disadvantages. Among them, the chromatography-mass spectrometry technology integrates the efficient separation ability of chromatography and the powerful analysis function of mass spectroscopy. On account of its characteristics of high sensitivity, wide dynamic range, good selectivity, and rich information, MSI has become the most commonly used analysis technology in metabolomics research and occupies an important position in the metabolomics analysis of biological samples, such as blood, urine, and cells (28). However, as for the analysis of metabolites in biological tissues or organs with a complex structure and high heterogeneity, the analytical method of chromatography-mass spectrometry technology has its limitations, as the pretreatment process of tissue homogenization and metabolite extraction, purification, and enrichment loses information on the spatial distribution of metabolites in the tissue. Therefore, to meet higher-level scientific research needs, spatial metabolomics has timely emerged.

Advances and developments in spatial metabolomics and MSI provide new opportunities for demonstrating a spectrum of tumors with metabolic heterogeneity, but also pose data mining challenges for the vast amount of qualitative, quantitative, and locational annotation information obtained from the metabolites in order to explore meaningful insights from high-dimensional spatial information. Theodore Alexandrov has reviewed the latest challenges of spatial metabolomics and MSI data mining through the perspective of a computational scientist and has listed tools and software packages for data analysis (29), such as METASPACE (30), MSiReader (31) and Cardinal packages (32). Moreover, it is noteworthy that artificial intelligence (AI) and machine learning have been successfully used to automated metabolite identification metabolomics *in vivo (* 33), and it is believed that in the near future they can also be applied to the identification of metabolite in MSI.

## 3 Spatial metabolomics combined multi-omics analysis

**Figure 2:**
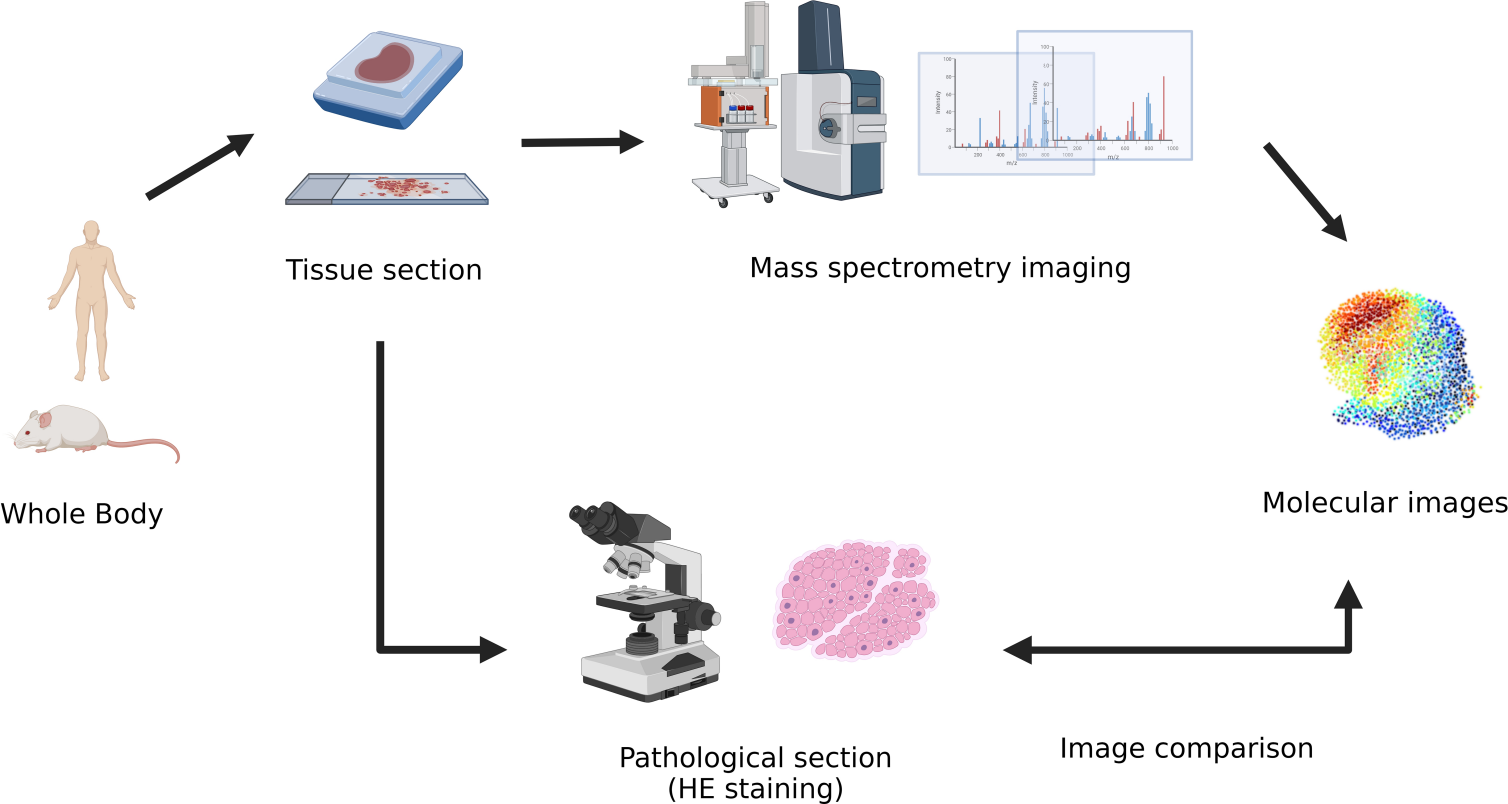
The process of transformation in multi-omics in the research of human tumor’s biology.

With the further application of spatial metabolomics, scientists have found it difficult to systematically and comprehensively analyze the regulatory mechanism of complex physiological processes through single-omics data. Hence, other perspectives, namely, spatial metabolomics combined with multi-omics analysis techniques, are required (**Figure 2**). Multi-omics joint analysis owns the advantages of accuracy, reliability, and depth (34), which can not only make up for the data problems caused by data noise and absence in a single omics analysis, but also reduce the false positive results caused by a single omics analysis through the mutual verification of multiple omics data resources. Therefore, multi-omics joint analysis is more conducive to systematically analyzing the multi-level mechanism or phenotype connection of biological models from different levels and from different perspectives, which can jointly explore the potential regulatory network mechanism within organisms, and provide more evidence for the mechanism of action of living organisms.

### 3.1 Spatial metabolomics combined with transcriptomics

The most common method of spatial metabolomics combined multi-omics analysis is spatial metabolomics combined transcriptomics (35). Transcriptome sequencing (36) yields a large number of differential genes and numerous regulatory networks in tissue samples, but it poses difficulties to identify critical pathways or key genes controlling them. Metabolites are the final embodiment of life activities, and the small changes in phenotypic traits will be amplified exponentially at the metabolic level. Therefore, we can use the metabolome to reflect the altered phenotype (37), but the separate metabolome detection cannot explain the genetic variation that affects the phenotypic changes. In short, spatial metabolomics combined with transcriptomics can be visually drawn from the gene transcript products (RNA) to various metabolites in the inferior metabolic pathways and can obtain the data of the spatial information simultaneously, thus exploring biological problems from two levels of both “cause” and “effect” and verifying each other. Generally speaking, spatial metabolomics combined with transcriptomics helps us to screen out key genes, marker metabolites, and regulatory metabolic pathways from the huge amount of detected data, deeply analyze the growth and development process of biological systems, and explain the complexity and integrity of biological processes. It provides important technical support in the study of tumorigenesis mechanism, metabolic reprogramming, and early diagnosis, and also proposes key ideas to solve the problem of studying the spatial and temporal heterogeneity of tumors.

D.H.Heiland et al. have deeply characterized glioblastoma in human neocortex slice models with the help of spatial transcriptomics, metabolomics and proteomics, and single-cell RNA sequencing (scRNA-seq) (38). By exploring the unique and common transcriptional programs between patients, they have inferred that glioblastoma develops along a defined neural lineage and adapts to inflammatory or metabolic stimuli, which makes them reminiscent of the reactive transformation of mature astrocytes. Metabolomics analysis and mass spectrometry imaging techniques combined analysis supports the hypothesis that tumor heterogeneity is determined by changes in the microenvironment. This suggests that glioblastoma cells adopt a transcriptional program that is similar to the inflammatory transformation of astrocytes, indicating that the overall changes in local native tumors and the tumor microenvironment collectively shape its transcriptional heterogeneity. It points out the forward directions for further studies of the metabolic reprogramming of glioma cells, and also provides critical data for adjusting the currently popular glioblastoma models.

Such problems often exist in actual scientific research processes, such as many genes and proteins are obtained after the differential comparative analysis, but their function seems to be not directly related to the phenotypic differences. Therefore, the significance of spatial metabolomics combined with multi-omics analysis is also reflected here. For the hot topics of the research field, a great number of reports have revealed the direct association of phenotypes with some key metabolites. And for emerging themes of the research fields, combined multi-omics analysis can provide a more panoramic network of molecular associations. Ultimately, among all the various differential omics molecules, the interrelated functional metabolites or pathways are selected for key discussion and research, to provide more directional guidance for the pioneers in this field.

### 3.2 Spatial metabolomics combined with lipidomics

Lipids are important biomolecules that play multiple roles in the cellular functions of mammalian organisms. Driven by the biological significance of lipids, lipidomics (39) is considered a key member of the multi-omics family to understand disease-specific dysfunction of lipid metabolism and lipid species associated with the disease, help to discover biomarkers and targets for monitoring therapeutic strategies, and to provide insights into tumor lipid analysis and pathophysiological mechanisms. Compared with normal cells, the lipid metabolic reprogramming of tumor cells can significantly affect the growth, proliferation, differentiation, apoptosis, and drug resistance of cancer cells, and it has been demonstrated in lung cancer and its subtypes (40). Lipidomics can detect differences in the abundance of lipids in different samples, but the differential spatial distribution of lipids cannot be obtained after the homogenization of tissue samples. Although spatial metabolomics detects few metabolites, it can sensitively obtain information on the spatial distribution of lipids. Spatial metabolomics combined with multi-omics analysis can complement the weaknesses and complement each other.

To more comprehensively reveal the distribution of anticancer drugs in tumor tissues in the process of cancer treatment and the drug’s impact on endogenous lipid metabolites, Yanyan Chen et al. first simulated the three-dimensional multicellular tumor spheroids with a complicated tumor microenvironment as a platform for *in vitro* research of drugs and endogenous metabolites. After finishing modeling, combined with MALDI-MSI and lipidomics, they studied the drug accumulation time and spatial distribution of hydroxychloroquine (HCQ) and its impact on lipid metabolism in lung cancer multicellular spheroids (41). The results show that using HCQ as treatment can significantly affect the changes in lipid abundance in multicellular spheroids of lung cancer, among which lipids like phosphatidyl ethanolamine (PE), phosphatidylcholine (PC), phosphatidylinositol (PI) can be more focused on. These findings can help us better understand the close relationship between lipid metabolic reprogramming caused by antitumor drugs and the tumor microenvironment (TME).

By using a comprehensive approach of global lipid metabolomics based on MSI as well as spatial metabolomics, Chao Zhao et al. investigated the toxic effects of bisphenol F (BPF) and its potential toxicology mechanisms in breast cancer xenografts and tumor metastasis-related tissues (e.g. liver and kidney) in breast cancer xenografts treated with BPF (42). Moreover, they used lipidomics and spatial metabolomics techniques to exploit and finally successfully apply a novel method for the fingerprint map analysis of global lipids and metabolites in the kidney and liver. The results have shown that exposure to BPF interferes with the metabolome and liposome of the liver and kidney, which results in metabolic reprogramming by activating amino acid biosynthesis and glycolysis metabolism in liver and kidney tissues. It also disrupts the biosynthesis and degradation of lipids, leading to abnormal cell membrane homeostasis and cell function. These observations provide greater insights into the critical role of metabolic reprogramming in the biological effects of BPF-induced tumor growth and proliferation.

Chenglong Sun et al. Have proposes an integrated spatially-resolved multiomics approach to explore cell-specific metabolic reprogramming and interactions in the microenvironment of gastric cancer (43). They have conducted Spatial metabolomics (SM), Spatial Lipidomics (SL) and Spatial Transcriptomics (ST) analysis on frozen cancer tissue sections from patients with gastric adenocarcinoma, and depicted spatially-resolved maps of metabolites, lipids, and gene expression patterns both in tumor cells and normal cells. They also have found that arginine and proline metabolism is significantly reprogrammed in gastric adenocarcinoma: arginine and its synthesis-associated gene *ASS1* are both upregulated in tumor sections. And the visualized results have shown that the expression of most lipids in gastric adenocarcinoma tissues is elevated except the expression of Phosphatidylserine (PS) and PI (44). It is notable that an immune desert phenotype has been found in both the stroma and parenchyma of gastric cancer tissues, indicating the lack of T cell tumors, which suggests that the alterations of relevant lipid in tumor tissues can strongly impact immune cell differentiation and activation (45).

Multi-omics analysis combined with spatial metabolomics can improve the integrity of biological information analysis and can visually detect gene, gene transcripts (RNA), various proteins that perform different biological functions, and even the various metabolites in the subordinating metabolic pathways. In conclusion, multi-omics analysis can help us expand our vision of the scientific field better and obtain a deeper understanding of the significance of omics interactions at different levels.

## 4 New progress and applications of spatial metabolism in head and neck tumors

Head and neck tumors are the sixth most common tumor worldwide (46), with a 5-year overall survival rate of 40% – 50%, of which head and neck squamous cell carcinoma (HNSCC) accounts for approximately 90% (47). HNSCC is a family of tumors derived from multiple sites, including the mouth, throat, pharynx, sinuses, and salivary glands, which occurs mainly in the area above the clavicle and before the cervical spine, and usually does not include intracranial, cervical, and ocular malignancies. To investigate the metabolic reprogramming mechanisms of head and neck tumors and improve the early diagnosis and corresponding treatment of it, extensive scientific research has been carried out around the world. Next, we will introduce the new progress in the research and application of spatial metabolomics in the metabolic mechanism of head and neck tumors.

### 4.1 Characterizing metabolic disorders of head and neck tumor

Energy metabolism reprogramming of tumor cells promotes rapid cell growth, proliferation, and differentiation by regulating energy metabolism. And it is considered a unique marker of tumor cells (48), including aerobic glycolysis, glutaminolysis, and upregulation of lipid synthesis. Among them, the famous scientist Otto Heinrich Warburg found the Warburg effect (49) in the 1920s. Even under the condition of sufficient oxygen supply, tumor cells prefer glycolysis rather than mitochondrial oxidative phosphorylation to generate energy, resulting in a high glucose uptake rate and elevated metabolic lactate content. Compared with traditional metabolomics, spatial metabolomics can help scientisits to provide relatively accurate metabolic evidence that drives the regulation of phenotype and to reflect physiological conditions at the spatial level well. Therefore, spatial metabolomics has surpassed traditional diagnostic methods that rely on a few diagnostic markers, and can effectively characterize tumor metabolic disorders (50), achieving precision medicine (51).

Metabolic reprogramming of glycolysis is an important aspect of tumor cells’ adaptation to hypoxia and is one of the marks of malignancy. Lingzhi Wang et al. have applicated together with LC-MS, metabolomics, immunohistochemical (IHC) (52) staining, and other analytical means for the research of the nasopharyngeal carcinoma (NPC) glucose metabolism process in depth. After the statistical analysis, they found that the tumor suppressor gene *CYLD* increases the stability and nuclear transportation of p53 by deubiquitination, to downregulate the transcription of phosphofructokinase-2/fructose-2, 6-Diphosphatase 3 (PFKFB3) (53), thus inhibiting the glycolysis process in NPC cells. The latest researches have shown that PFKFB3 has been associated with various aspects of cancer, including cancer cell proliferation, vascular invasiveness, drug resistance, and the TME (54). Furthermore, Baoyu He et al. have identified and functionally characterized a novel, metabolically relevant, long, non-coding RNA (lncRNA) LINC00930 (55). The result suggests that LINC00930 promotes the glycolysis process in NPC cells by up-regulating the expression of PFKFB3 in NPC. Therefore, the lack of expression of *CYLD* enhances the reprogramming of glucose metabolism and tumor progression and is associated with poor prognosis in NPC through PFKFB3 upregulation. These conclusions suggest that *CYLD* can be used as a clinical biomarker for predicting NPC prognosis, and that PFKFB3 may also be a new therapeutic target for targeted therapy in the future (56).

### 4.2 Diagnosis and classification of head and neck tumors

Early tumor diagnosis is essential to prolong patients’ survival time and improve their quality of life and prognosis (57). The traditional methods of early tumor diagnosis mainly include imaging examination, blood and tumor marker detection, pathological puncture biopsy, and cell smear examination. However, due to tumor heterogeneity, it is difficult for these methods to have both high specificity and sensitivity. After the diagnosis of tumors, accurate and timely classification and grading not only influence the survival time of patients, but also play a decisive role in the selection of treatment options (58).

With the continuous research on the mechanism of tumor metabolic reprogramming, scientists have found that metabolic reprogramming of tumor cells is often driven by a variety of signaling pathways and transcription factors (59). Therefore, screening of key protein markers of tumor-dependent metabolic reprogramming has a promising prospect, which is conducive to the realization of early clinical diagnosis and the development of novel drugs targeting tumor metabolism (60).

According to the latest application of spatial metabolomics in the diagnosis and classification of head and neck tumors, Luojiao Huang et al. have applied spatial metabolomics analysis by using AFADESI-MSI technology to thyroid tumors (61). Firstly, they characterized high-resolution, tumor region-specific metabolites spectra for each pathologic type of thyroid tumors. And then they identified 83 sets of metabolic biomarkers which could visually differentiate benign follicular adenoma (FA) from differentiated thyroid cancers including papillary thyroid carcinoma (PTC) and thyroid follicular cancer (FTC). Finally, a molecular diagnostic strategy was established to distinguish between 65 pathologic thyroid tumors. Overall, the diagnostic models based on the metabolic spectrum of different types of thyroid tumors, have showed a prediction accuracy of 83.3%. For the rarer and more aggressive medullary thyroid cancer (MTC), Andrew Smith et al. have analyzed tissue samples from 7 patients with MTC with the MALDI-MSI technology (62). After performing a proteomic combination analysis, they have identified and selected several potential tumor markers associated with the pathogenesis of MTC. These findings can provide a valuable starting point for further studies on the diagnosis and analysis of thyroid tumors in the preoperative stage.

### 4.3 Targeted therapy for head and neck tumors

Tumor-targeted therapy is defined as the use of drugs that target different cancer cell targets and do not cause simultaneous harm to non-cancer cells as conventional chemotherapeutic drugs do (63). The purpose of investigating targeted therapy is to discover and attack specific areas or substances in tumor cells or to detect and prevent specific signaling molecules that are sent within the tumor cells that command the growth of tumor cells. Nowadays, tumor-targeted therapy is widely used in non-small cell lung cancer (NSCLC) (64, 65), triple-negative breast cancer (BC) (66), and other cancers. But deeper research of targeted therapy for head and neck tumors is still in great need.

Recently, amino acid metabolic reprogramming in head and neck tumor is attracting increasing attention, especially in glutamine metabolism. Glutamine, despite being a non-essential amino acid, is the most abundant amino acid in circulation (67). In the normal human body, the content of glutamine in the blood is relatively constant, but when the cells are under extremely vigorous catabolic conditions, the decomposition of glutamine is significantly elevated (68). Toshimitsu Ohashi et al. have combined capillary electrophoresis-mass spectrometry imaging (CE-MS) with metabolomics to comparatively analyze 23 HNSCC patients and 6 patients without cancer. They have proved that glutamine metabolism was markedly up-regulated in HNSCC tissues, and glutamine metabolites, such as glutathione (GSH), were accumulated in HNSCC tissues (69), to mediate the redox balance of HNSCC tumor cells, and to meet the exuberant needs for energy, anabolic carbon sources, and nitrogen sources of tumor cells. The above results indicate that the up-regulation of glutamine metabolism plays an important role in autophagy regulation in HNSCC tumor cells through the homeostasis of reactive oxygen species free radicals (ROS). In the future, we can also continue to explore anti-tumor drugs targeting glutamine metabolism.

Additionally, the reprogramming of lipid metabolism in head and neck tumors is also being further researched by scientists around the world. Normal body cells rely mainly on the free fatty acids from the food to synthesize the lipid components. Due to the vigorous metabolism of tumor cells, they need a large amount of lipid supply. Therefore, tumor cells depend more on *de novo* fatty acid synthesis to meet their special lipid needs (70), thus undergoing lipid metabolism reprogramming (71). Schmidt J et al. have used the MALDI-MSI technology to search proteins and lipids that are significantly overexpressed in tumor tissues from numerous HNSCC samples (72). They have found that in the visible low-molecular-weight proteins of this laryngeal cancer patient specimen, S100A8 and S100A9 are highly expressed in the tumor tissue region, but not in the surrounding healthy tissue. In addition, they also found that lysophosphatidylcholine (LPC) levels were significantly decreased, even almost completely abolished in the tumor region, while the glycerophospholipid PE-P and PC accumulated significantly. Further research by Kerkhoff C et al. has found LPC-digested lysophospholipase 1 (Human Acyl-protein thioesterase 1, LYPLA1) (73) accumulation in the tumor region, suggesting that the progression of HNSCC *in vivo* may depend on lysophospholipid (LP) supply. These findings have confirmed that the hypoxia-induced fatty acids uptake and lipid droplet accumulation of tumor cells constitute lipid metabolism reprogramming, which can effectively prevent the toxicity caused by ROS accumulation, thus providing higher survival benefits and treatment resistance for tumor tissues. Furthermore, it is of great significance to the research of HNSCC metabolic mechanisms and provides targets that can be further explored for the treatment of HNSCC, which has a broad clinical application prospect.

As an important component of personalized cancer therapy, novel drugs that target to tumor metabolism have progressed from preclinical studies to clinical trials, and in some cases have shown great efficacy. It is believed that with the advancement of drug metabolism analytical technology, researchers will be able to discover more new drug targets, construct appropriate patient models and accurately identify target patients, so as to provide patients with more drug choices and to better improve patients’ survival rate.

## 5 Conclusions and outlook

In 1927, Otto Heinrich Warburg discovered the Warburg effect and proposed the concept of metabolic reprogramming of tumor cells, which made the research on tumor metabolism the focus topic in tumor research today. Tumor metabolic reprogramming is one of the important features of malignancy, and aerobic glycolysis is one of the most typical features. In this review, we firstly summarized the development and challenges of spatial metabolomics, and the latest researches of spatial metabolomics combined multi-omics analysis in the metabolic mechanisms of tumor. On the basis, we reviewed the novel researches and applications of metabolic reprogramming in head and neck tumors with the technology of spatial metabolomics. Finally, we made an outlook of it to the future.

Firstly, we should understand that the regulation of gene expression is one of the main sources of genetic variation, transcription predicts gene function and reveals the sequence of a gene that can be a cis-acting element, proteins are the executors of biological function, metabolism is the description of a phenotype, and morphology is the concrete presentation of regulatory results. Only through the systematic combination of genomic and phenomic data, can we truly realize the closed loop of big data for biological systems. In biological tissues, the global analysis of non-functional metabolites with spatial distribution is key to understanding biomolecular processes (5). The diversity and complexity of tumor tissues and the ubiquity of tumor metabolic heterogeneity have made tumor metabolic research face a large dilemma. Therefore, the use of spatial information to characterize tumor metabolism facilitates our understanding of complex cancer metabolic reprogramming, the discovery of certain metabolites as biomarkers for early diagnosis and classification, and the identification of potential metabolic vulnerabilities that may be targeted for tumor comprehensive therapy. Compared with the traditional biological information statistical method, spatial metabolomics technology is beginning to enter the eyesight of scientists. It integrates the technical characteristics of MSI and metabolomics. And it can combine multiple omics to carry out a comprehensive analysis, with the features of wide coverage, high sensitivity, wide dynamic range, high specificity, and high heterogeneity, which can provide visualization results of the spatial and temporal distribution of metabolites for the study of tumor metabolic mechanism.

Up to now, the unique position of spatial metabolomics in the field of tumor research has been widely recognized, and it has begun to be applied in the study of metabolic mechanisms of human tumors and new drug development. In addition to these studies mentioned above, Jian Shen et al. have evaluated the response of NSCLC patients to neoadjuvant therapy using spatial metabolomics (74) to judge the prognosis of patients better. Judith Martha Neumann et al. have used histology-guided spatial metabolomics to detect the differences between the subtypes of NSCLC and to distinguish tumor regions and stroma regions, which has contributed to the analysis of each subtype of NSCLC, hence, promoting the development of therapeutic strategies (75). In conclusion, spatial metabolomics helps to reveal what has happened in tumor cells at the molecular level, providing further insight into our understanding of metabolic reprogramming in tumor cells. Spatial metabolomics, as a breakthrough technology, brings molecular diagnosis numerous new opportunities. However, it also faces different challenges, such as the identification and chromatographic separation of metabolites and mass spectrometry database and data sharing issues (76). Fortunately, with advances in instrumentation, experimental techniques, and analytical software, many challenges of spatial metabolomics have been alleviated.

At present, spatial metabolomics has shown a vigorous development trend in the study of metabolic mechanisms of human tumors, yet new applications and researches remain underdeveloped in the field of head and neck tumors. Therefore, further research is warranted. We have gained many valuable lessons from research on the metabolism of other human tumors with the help of spatial metabolomics technology, which offers important guidance to our otolaryngology head and neck surgeons to study the metabolic mechanism of head and neck tumors. Combined with IHC analysis and scRNA-seq of head and neck tumors previously conducted by our research group (77), we can also conduct in-depth statistical analysis and research on tumor metabolism of HNSCC and NPC. By combining spatial metabolomics with multi-omics analysis and other metabolomics, we can carry out mutual verification among multi-omics data sets and systematically analyze the relationship between gene expression and the phenotype in head and neck tumors from different levels and perspectives, to further explore the regulatory network of the pathogenesis of head and neck tumors *in vivo*, and to provide more visual evidence for the specific manifestation and intrinsic root of tumor metabolic reprogramming. Additionally, we are expecting to discover more sensitive biomarkers for the early diagnosis of head and neck tumors, and also to discover novel targets for drug-targeted therapy. If we can combine spatial metabolomics technology and robot surgery technology into the clinical routine workflow furtherly, through the study of a large number of clinical data to screen the appropriate tumor markers, it can be more efficient, sensitive, and accurate in early diagnosis, classification diagnosis and intraoperative margin analysis of head and neck tumors. Furthermore, it can even meaningfully instruct oncology therapy, hence better prolonging patients’ survival time and improving their long-term quality of life.

## Author contributions

YZ: literature search, visualization, writing - original draft preparation. CL: investigation, supervision, revised the manuscript. YC: data analysis, investigation, supervision. HD and SG: funding acquisition, project administration. ZS: idea for the review, conceptualization, writing - reviewing and editing, project administration, funding acquisition. All authors wrote one part or several parts of the manuscript. All authors contributed to conceptualising and writing the manuscript. All authors read and approved the final manuscript. All authors contributed to the article and approved the submitted version.
